# Exercise-Induced Bronchoconstriction in School-Aged Children Born Very Preterm: Prevalence and Associated Clinical Factors

**DOI:** 10.3390/jcm15186995

**Published:** 2026-09-10

**Authors:** Ercan Yılmaz, Nezihe Koker Ozer, Hatice Turgut, Erdem Topal, Ramazan Özdemir, Recep Gunakın, Mehmet Aslan

**Affiliations:** 1 Faculty of Medicine, Department of Pediatric Allergy and Immunology, Inonu University, Malatya 44280, Turkey; 2 Faculty of Medicine, Department of Neonatology, Inonu University, Malatya 44280, Turkey; 3 Faculty of Medicine, Department of Pediatric, Inonu University, Malatya 44280, Turkey

**Keywords:** exercise-induced bronchoconstriction, preterm, prevalence, school-aged children

## Abstract

**Background:** Children born preterm may have persistent alterations in airway function that extend into school age; however, exercise-induced bronchoconstriction (EIB) in this population remains insufficiently characterized. This study aimed to determine the prevalence of EIB in school-aged children born at ≤32 weeks of gestation and to investigate associated clinical and neonatal factors. **Methods:** This single-center cross-sectional study included 42 children born at ≤32 weeks of gestation who were evaluated at 7–8 years of age. Perinatal, neonatal, respiratory, and atopic characteristics were obtained from medical records and parental reports. Baseline spirometry was performed before a standardized treadmill exercise challenge. Spirometry was repeated at 5, 10, 15, and 30 min after exercise. EIB was defined as a ≥10% decrease in forced expiratory volume in one second (FEV_1_) from baseline at any post-exercise time point. **Results:** The median gestational age was 29 (25–32) weeks, and 31% had a history of bronchopulmonary dysplasia. EIB was identified in 13 of 42 children (31%). Post-exercise EIB positivity was observed at 5, 10, 15, and 30 min in 9.5%, 16.7%, 11.9%, and 9.5% of participants, respectively. Dyspnea was the most frequent exercise-related symptom (38%). No significant differences were observed between EIB-positive and EIB-negative children regarding gestational age, birth weight, bronchopulmonary dysplasia, neonatal respiratory support, baseline pulmonary function, total IgE, or peripheral eosinophil count. **Conclusions:** EIB was detected in approximately one-third of school-aged children born at ≤32 weeks of gestation. These findings suggest that clinically relevant exercise-related airway hyperresponsiveness may persist into school age despite relatively preserved baseline spirometry.

## 1. Introduction

Preterm birth is a significant public health issue, affecting approximately 10 per cent of live births worldwide and remaining one of the leading causes of neonatal mortality and long-term childhood morbidity [1,2]. In recent years, survival rates for extremely preterm infants in particular have increased significantly, thanks to antenatal corticosteroid treatments, surfactant therapy, advanced mechanical ventilation strategies and advances in neonatal intensive care services [2,3]. However, these rising survival rates have led to health issues in individuals born prematurely becoming more apparent, not only during the neonatal period but also throughout childhood and adulthood. Consequently, preterm birth is now regarded not merely as a condition affecting the early postnatal period, but as a chronic developmental process with potential lifelong effects [3,4].

A significant proportion of lung development takes place during the final trimester of pregnancy. Preterm birth can disrupt the critical period of alveolarization and pulmonary vascular development, leading to permanent structural changes in the airways and lung parenchyma [4,5]. Furthermore, factors associated with intensive care, such as mechanical ventilation, oxygen therapy, inflammation and neonatal infections, can also exacerbate damage to the developing lungs [5,6]. The concept of ‘new broncho-pulmonary dysplasia’, as defined today, is characterized by alveolar and vascular developmental abnormalities rather than classic fibrotic lung disease, and is recognized as one of the key mechanisms underlying lifelong respiratory dysfunction in individuals born prematurely [5,6].

Studies examining long-term lung function in children born prematurely show that these individuals may develop reduced FEV_1_ and FEV_1_/FVC ratios, peripheral airway dysfunction, reduced lung reserve and obstructive respiratory dysfunction that may persist into adulthood [4,7,8,9]. It has been shown that these changes are not limited solely to cases in which broncho-pulmonary dysplasia (BPD) develops; lower lung function has also been reported in preterm infants without a diagnosis of BPD compared with their term peers [7,8,9]. However, the long-term respiratory outcomes of preterm infants are highly heterogeneous and cannot be explained by gestational age or the severity of neonatal illness alone. It is thought that genetic predisposition, prenatal inflammation, postnatal lung growth and environmental exposures all play a role in the development of these different phenotypes [4,8].

Exercise-induced bronchoconstriction (EIB) is defined as a temporary narrowing of the airways following intense physical activity and is one of the major causes of symptoms such as cough, dyspnea, wheezing and a feeling of tightness in the chest in children [10,11]. EIB can occur not only in individuals with asthma but also in children without asthma and in those born prematurely [10,11,12]. In its pathophysiology, osmotic changes resulting from airway drying and cooling due to increased ventilation during exercise, along with the release of inflammatory mediators, play a key role [10]. In preterm infants, it is thought that immature airways, changes in alveolar structure and early-life lung damage contribute to this response [4,5].

Although there are numerous studies assessing exercise capacity in preterm infants, studies investigating the prevalence of EIB using an objective exercise provocation test are quite limited [4,11,13]. Furthermore, due to the use of different exercise protocols, varying diagnostic criteria and heterogeneous patient groups in existing studies, there are significant differences between the reported prevalence figures [11,13]. Furthermore, the role of neonatal risk factors such as gestational age, birth weight, bronchopulmonary dysplasia, mechanical ventilation or oxygen therapy in the development of EIB remains unclear [4,8,9]. Systematic reviews published in recent years and the ATS’s guidelines on post-prematurity respiratory disease emphasize the importance of long-term respiratory follow-up in school-aged preterm children; however, they note that there is insufficient evidence to determine which children would benefit from further functional assessment [4,8,14].

The aim of this study was to determine the prevalence of exercise-induced bronchoconstriction in school-aged preterm children using a standard treadmill exercise provocation test, and to assess the relationship between the development of EIB and demographic characteristics, neonatal risk factors, a history of atopic disease, laboratory findings and baseline respiratory function tests.

## 2. Materials and Methods

### 2.1. Study Design and Population

This single-center, cross-sectional observational study was conducted at the Clinic of Pediatric Immunology and Allergic Diseases, Turgut Ozal Medical Center, Inonu University. Following ethics committee approval, potentially eligible children were identified from the electronic records of the Inonu University Faculty of Medicine Neonatal Intensive Care Unit (NICU), where they had been followed during the neonatal period. Initial eligibility criteria were birth at ≤32 weeks of gestation, age 7–8 years at the time of assessment, and availability of perinatal and neonatal medical records. Children with an acute respiratory tract infection or a respiratory tract infection within the preceding four weeks, a serious neuromotor or orthopedic condition precluding exercise, hemodynamically significant congenital heart disease, or another medical contraindication to exercise testing were not eligible to proceed with testing. Because all assessments were conducted during a single study visit, children who were not clinically eligible for exercise testing on the day of assessment were not rescheduled or reassessed for study purposes and were excluded from the final analysis.

A total of 192 children were identified from the NICU records. Parents were contacted by telephone using the contact information available in the hospital records. Among those successfully contacted, 66 agreed to participate and attended the Pediatric Allergy Outpatient Clinic for assessment. Following clinical eligibility assessment, baseline spirometry and exercise challenge testing were attempted as appropriate. All clinical assessments and pulmonary function testing were performed during a single outpatient visit.

**Figure 1:**
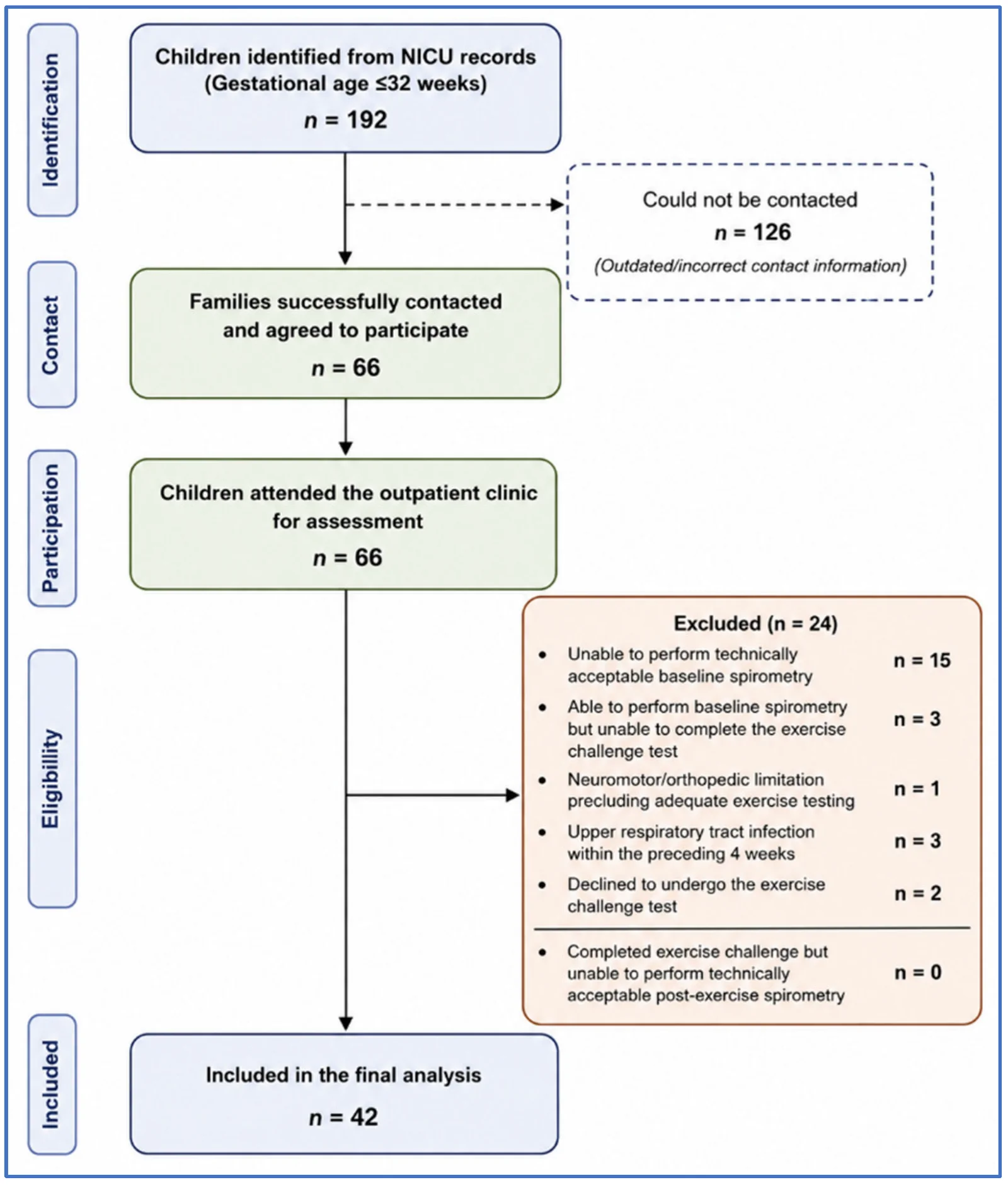
Study Flow Diagram.

Of the 66 children who attended the study visit, 24 were excluded from the final analysis: 15 were unable to perform technically acceptable baseline spirometry, 3 successfully performed baseline spirometry but were unable to complete the exercise challenge test, 1 had an orthopedic limitation precluding adequate exercise testing, 3 had experienced an upper respiratory tract infection within the preceding four weeks, and 2 declined to undergo the exercise challenge test. No child who completed the exercise challenge was subsequently excluded because of technically unacceptable post-exercise spirometry. Consequently, 42 children completed the required testing and were included in the final analysis (Figure 1). All clinical assessments, spirometry and exercise provocation tests were carried out on the same day and during a single visit.

### 2.2. Demographic, Perinatal and Clinical Data

Detailed demographic and clinical characteristics of all participants were recorded. Age, sex, family history, respiratory history (including previous hospitalization due to lower respiratory tract infection), concomitant atopic diseases, family history of atopic diseases, history of breastfeeding, and pet ownership were assessed. Patients’ height and weight measurements were taken using standard methods, and height z-scores and weight z-scores were calculated according to age and gender. Anthropometric assessments were carried out in accordance with national reference data [15].

Perinatal and neonatal data were obtained from neonatal intensive care unit (NICU) records and hospital electronic medical records. The variables collected included gestational age, birth weight, small for gestational age (SGA), neonatal sepsis, neonatal apnea, bronchopulmonary dysplasia (BPD), patent ductus arteriosus (PDA), intraventricular hemorrhage (IVH), and necrotizing enterocolitis (NEC). Information regarding surfactant administration, the requirement for mechanical ventilation and continuous positive airway pressure (CPAP), the duration of mechanical ventilation and oxygen therapy, and length of NICU stay was also recorded.

Small for gestational age was defined as a birth weight below the 10th percentile for gestational age and sex. Bronchopulmonary dysplasia and other neonatal morbidities were identified according to the diagnoses documented by the attending neonatologists during the neonatal period.

Comorbid conditions, including attention-deficit/hyperactivity disorder, visual impairment, hearing impairment, and motor developmental disorders, were recorded from medical records and parental reports. These conditions were not reassessed during the study visit; only previously established specialist diagnoses were considered.

### 2.3. Laboratory Measurements

Peripheral venous blood samples obtained during the study visit or available from recent hospital records were used to determine absolute peripheral blood eosinophil counts and total immunoglobulin E (IgE) levels. Absolute eosinophil counts were expressed as ×10^3^/mm^3^ and total IgE concentrations as IU/mL.

### 2.4. Respiratory Function Tests

Prior to the exercise provocation test, all participants underwent spirometry in accordance with the recommendations of the American Thoracic Society (ATS) and the European Respiratory Society (ERS) [16]. In the spirometric assessment, forced expiratory volume in the first second (FEV_1_), forced vital capacity (FVC), FEV_1_/FVC ratio, mid-expiratory flow rate (FEF25–75) and peak expiratory flow (PEF) values were recorded. At least three acceptable and reproducible maneuvers were obtained for each participant, and the highest values were used in the analysis. Results were expressed as percentages of the predicted values (% predicted) according to age, sex and height.

### 2.5. Exercise Provocation Test

The exercise provocation test was conducted in accordance with the guidelines on exercise-induced bronchoconstriction published by the American Thoracic Society [10]. All participants exercised on a treadmill, aiming to reach approximately 85–90% of their age-predicted maximum heart rate. The target exercise intensity was maintained for at least 6 min. Exercise testing was performed under standardized environmental conditions in an indoor laboratory, with an ambient temperature of 20–25 °C and relative humidity of <50%, in accordance with ATS recommendations [10]. To minimize potential pharmacological influences on airway responsiveness, participants were instructed to withhold medications before testing in accordance with ATS recommendations, including short-acting bronchodilators for at least 8 h, long-acting bronchodilators for at least 48 h, leukotriene receptor antagonists for at least 24 h, and other medications that could potentially influence airway responsiveness whenever clinically appropriate. Spirometric measurements were repeated at 5, 10, 15 and 30 min post-exercise. A decrease of 10% or more in FEV_1_ relative to the baseline value at any time point post-exercise was considered exercise-induced bronchoconstriction.

The change in FEV_1_ following exercise (ΔFEV_1_) was calculated as a percentage change relative to the baseline value. Symptoms such as dyspnea, cough, wheezing and chest tightness that arose during or after the test were recorded. Patients requiring bronchodilator treatment following the test were also recorded.

### 2.6. Statistical Analysis

Statistical analyses were performed using IBM SPSS Statistics for Windows, version 25.0, IBM Corp., Armonk, NY, USA. The normality of continuous variables was assessed using visual methods and the Shapiro–Wilk test. Normally distributed continuous variables were presented as mean ± standard deviation, whilst non-normally distributed variables were presented as median and minimum–maximum. Categorical variables were expressed as counts and percentages. In comparisons between the EIB-positive and EIB-negative groups, the independent samples Student’s *t*-test was used for normally distributed continuous variables, and the Mann–Whitney U test was used for non-normally distributed variables. Categorical variables were compared using the Pearson chi-square test or, where the expected cell counts were low, Fisher’s exact test. All tests were conducted as two-tailed tests, and a *p*-value of <0.05 was taken as the threshold for statistical significance.

## 3. Results

### 3.1. Study Population and Demographic Characteristics

The study included a total of 42 preterm-born children assessed at the age of eight. Twenty-seven of the participants (64.2%) were girls, and the median age was 7 (7–8) years. The median height z-score was −0.08 (−1.82–2.18), whilst the median weight z-score was −0.36 (−2.40–2.18). A history of consanguineous marriage among the parents was present in 11 (26.2%) of the children. The median gestational age of the preterm children was 29 (25–32) weeks, and the median birth weight was 1.23 (0.75–2.35) kg. Eight children (19%) were born small for gestational age (SGA). Regarding the mode of delivery, 41 children (97.6%) were born by Caesarean section and 1 (2.4%) by vaginal delivery. Respiratory distress syndrome (RDS) and congenital pneumonia were reported in 9 (21.4%) and 11 (26.2%) children, respectively, and 19 (45.2%) had received antenatal corticosteroids. The most common morbidity in the neonatal period was neonatal sepsis, which was detected in 28 (66.7%) children. This was followed by premature infant apnea (*n* = 17, 40.5%) and the need for mechanical ventilation (*n* = 17, 40.5%). Bronchopulmonary dysplasia (BPD) was observed in 13 (31%), patent ductus arteriosus (PDA) in 8 (19%), intraventricular hemorrhage (IVH) in 5 (11.9%) and necrotizing enterocolitis (NEC) in 2 (4.8%) children.

During their stay in the neonatal intensive care unit, 17 (40.5%) of the infants received mechanical ventilation, 34 (81%) received CPAP treatment, and 9 (21.4%) were administered surfactant. The median duration of mechanical ventilation was 1 (1–7) days, the median duration of oxygen therapy was 3 (2–61) days, and the median length of stay in the neonatal intensive care unit was 44 (3–93) days. Four children (9.5%) had previously been hospitalized due to a lower respiratory tract infection. Furthermore, 23 children (54.8%) had been breastfed for more than six months, and it was reported that 2 children (4.8%) had pets at home.

On examination of personal atopic history, 5 children (11.9%) had a history of asthma, 6 (14.3%) had a history of allergic rhinitis, 3 (7.1%) had a history of eczema and 2 (4.8%) had a history of food allergy. In terms of family history, asthma was reported in 6 (14.3%), allergic rhinitis in 14 (33.3%) and eczema in 7 (16.7%) children. When assessing comorbid health conditions, attention deficit hyperactivity disorder (ADHD) was identified in 10 (23.8%) children, visual impairment in 9 (21.4%) children, motor development disorders in 7 (16.7%) children, and hearing impairment in 3 (7.1%) children. In the laboratory assessment, the median total IgE level was 58.4 (5.7–552) IU/mL, whilst the median eosinophil count was 0.23 (0.10–0.85) × 10^3^/mm^3^. The children’s demographic, perinatal and clinical characteristics are summarized in Table 1.

To assess the representativeness of the final sample, perinatal and neonatal characteristics were compared between children included (*n* = 42) and not included (*n* = 150) in the final analysis. No significant differences were observed in sex, gestational age, birth weight, 5 min Apgar score, SGA, neonatal sepsis, necrotizing enterocolitis, PDA, IVH, apnea of prematurity, BPD, mechanical ventilation requirement or duration, or length of NICU stay (all *p* > 0.05; Supplementary Table S1).

### 3.2. Exercise Challenge Test Findings

In preterm infants who underwent an exercise provocation test, the mean results of the baseline pulmonary function test were FEV_1_ 89.19 ± 8.48%, FVC 82.69 ± 7.83%, FEV_1_/FVC 108 ± 7.63%, FEF25–75 101.92 ± 23.02% and PEF 85.80 ± 14.93%. As a result of the exercise provocation test, exercise-induced bronchoconstriction (EIB) was detected in 13 (31%) children. EIB positivity was observed in 4 (9.5%) children at the 5th minute, 7 (16.7%) at the 10th minute, 5 (11.9%) at the 15th minute and 4 (9.5%) at the 30th minute of the test. ΔFEV_1_ values were calculated as 5.00 ± 5.93%, 5.28 ± 6.12%, 3.57 ± 7.84% and 3.21 ± 7.82% at the 5th, 10th, 15th and 30th minutes, respectively. The most common symptom during the exercise test was dyspnea, which was observed in 16 (38%) children. This was followed by cough (*n*: 5, 11.9%), wheezing (*n*: 2, 4.8%) and a feeling of tightness in the chest (*n*: 1, 2.4%). Following the test, 4 (9.5%) children were treated with a bronchodilator (Table 2).

### 3.3. Comparison of Children with and Without Exercise-Induced Bronchoconstriction

Thirteen (31%) children who tested positive for EIB following an exercise provocation test were compared with 29 (69%) children who tested negative for EIB. No significant differences were observed between the two groups in terms of age, sex, gestational age, birth weight, or 5 min Apgar score (all *p* > 0.05). Similarly, no statistically significant differences were found between the groups in terms of bronchopulmonary dysplasia (BPD), neonatal apnea, the need for mechanical ventilation, duration of mechanical ventilation, duration of oxygen therapy and length of stay in the neonatal intensive care unit (all *p* > 0.05). Furthermore, a history of breastfeeding for more than six months, a diagnosis of asthma and a family history of asthma were also similar between the groups. When baseline respiratory function tests were evaluated, although baseline FEV_1_ and FEF25–75 values were higher in the EIB-positive group, these differences did not reach statistical significance (*p* = 0.054, *p* = 0.060). No significant differences were observed between the groups in terms of total IgE levels and peripheral blood eosinophil counts (*p* = 0.336 and *p* = 0.331, respectively) (Table 3).

## 4. Discussion

Approximately one-third (31%) of school-aged children born preterm exhibited exercise-induced bronchoconstriction (EIB), yet no conventional clinical or neonatal characteristics distinguished affected children from those without EIB. Neither perinatal risk factors nor baseline pulmonary function measurements were associated with the presence of EIB, highlighting the limited ability of routine clinical assessment to identify children with exercise-induced airway dysfunction. These findings indicate that standardized exercise challenge testing offers incremental diagnostic value by revealing latent airway hyperresponsiveness that is not apparent at rest and may therefore improve the long-term respiratory evaluation of children born preterm.

Although long-term respiratory dysfunction in individuals born prematurely is well established [17], the number of studies assessing the prevalence of EIB using objective exercise provocation tests is quite limited. The majority of existing studies have focused on baseline spirometry, cardiopulmonary exercise capacity or bronchial hyperreactivity, whilst the assessment of EIB in school-aged preterm children using serial FEV_1_ measurements following a standard exercise test has rarely been performed [4,8,9,11]. The 31% prevalence of EIB identified in our study suggests that clinically undetected airway hyperreactivity, which manifests during physical activity, may be more common in preterm children than previously thought. This finding supports the need to assess preterm children not only in terms of their resting lung function but also in terms of their functional exercise response.

The effects of preterm birth on lung development are not limited to structural damage observed solely during the neonatal period. Early disruption of alveolarization, impaired pulmonary vascular development and airway remodeling can lead to functional changes that persist throughout childhood and into adulthood [5,8,9,11]. With the emergence of the concept of ‘new broncho-pulmonary dysplasia’ in recent years, it has become clear that premature lung disease is characterized by alveolar developmental delay and heterogeneous lung growth rather than fibrosis [5]. Consequently, long-term respiratory function may vary significantly even amongst children with the same neonatal history who were born prematurely. Our findings also support this view and suggest that the airway response during exercise may be influenced by individual lung development and postnatal remodeling processes.

One of the most noteworthy findings of the present study was the absence of significant associations between EIB and neonatal risk factors. Lower gestational age, very low birth weight, bronchopulmonary dysplasia (BPD), prolonged mechanical ventilation, and extended oxygen supplementation have traditionally been regarded as major determinants of adverse long-term respiratory outcomes in individuals born preterm [2,3,18]. Numerous longitudinal cohort studies have demonstrated that these neonatal factors are associated with impaired lung growth, persistent airflow limitation, and reduced expiratory airflow extending into adolescence and adulthood [2,3]. Consequently, it might be expected that children with greater neonatal respiratory morbidity would also exhibit a higher prevalence of exercise-induced bronchoconstriction. However, our findings did not support this assumption.

This observation is consistent with emerging evidence indicating that long-term respiratory outcomes after preterm birth are considerably more heterogeneous than previously recognized. Recent reviews emphasize that respiratory trajectories diverge substantially during childhood, with some individuals demonstrating preserved lung growth and near-normal pulmonary function despite severe neonatal respiratory disease, whereas others develop persistent airway dysfunction despite relatively uncomplicated neonatal courses [2,18]. These heterogeneous trajectories likely reflect the complex interaction of multiple postnatal influences rather than the isolated effects of neonatal disease severity alone. Continued alveolarization and airway growth throughout childhood, genetic susceptibility, epigenetic programming, respiratory viral infections, environmental tobacco smoke exposure, air pollution, physical activity, and other environmental factors may all contribute to remodeling of the developing airways and ultimately determine respiratory phenotype in later life [2,9,18].

Importantly, EIB reflects dynamic airway hyperresponsiveness rather than fixed airflow limitation. Although both conditions may coexist, the biological mechanisms underlying exercise-induced airway narrowing are only partially explained by structural lung injury sustained during the neonatal period. Airway epithelial dysfunction, persistent low-grade inflammation, altered airway smooth muscle responsiveness, autonomic dysregulation, and impaired airway repair have all been proposed as contributors to exercise-induced airway hyperresponsiveness in children born preterm [9,18]. Consequently, neonatal respiratory morbidity alone may be insufficient to predict which children will subsequently develop EIB.

An additional consideration is the relatively modest sample size of the present study, which may have reduced statistical power to detect weak or moderate associations between neonatal exposures and later EIB. Therefore, the absence of significant relationships should not necessarily be interpreted as evidence that neonatal factors have no influence on subsequent exercise-induced airway dysfunction. Rather, our findings suggest that EIB is likely determined by a multifactorial process in which neonatal characteristics represent only one component of a much broader developmental pathway. From a clinical perspective, these results indicate that neonatal history alone may not provide adequate risk stratification for exercise-induced airway dysfunction, supporting the use of objective exercise challenge testing during follow-up of school-aged children born preterm.

The most clinically significant message of our study is that, although EIB is observed with considerable frequency in school-aged children born prematurely, it can easily be overlooked in routine clinical assessments. In daily practice, most of these children are considered to be at low respiratory risk as long as their baseline spirometry results are normal. However, our findings indicate that significant bronchoconstriction can develop during exercise even in children with normal baseline lung function. Therefore, a standard exercise provocation test should be included in the diagnostic assessment, particularly in preterm children presenting with symptoms such as dyspnea, cough, reduced performance or avoidance of physical activity during exercise. Early diagnosis and appropriate treatment are important for maintaining physical activity, preserving quality of life and preventing unnecessary restrictions on exercise.

The strengths of this study include the evaluation of all participants at the same school age, the availability of detailed neonatal records, and the use of a standardized exercise challenge protocol performed at a single center by the same study team. Nevertheless, several limitations should be acknowledged. First, the single-center design and relatively modest sample size may have limited the statistical power to detect associations between EIB and neonatal risk factors. Therefore, the absence of significant relationships with perinatal and neonatal characteristics should be interpreted cautiously, as modest effects may have remained undetected. As there was no control group of healthy term-born infants, the prevalence of EIB could not be directly compared with that in the general population. Furthermore, the absence of supplementary assessments, such as FeNO or cardiopulmonary exercise testing, limits mechanistic interpretations. An important limitation is the potential for selection bias, as only 42 of the 192 children initially identified from NICU records were included in the final analysis, primarily because many families could not be contacted several years after neonatal discharge. Although no significant differences were observed in the available perinatal and neonatal characteristics between included and non-included children, selection bias related to unmeasured post-discharge factors cannot be excluded. In addition, exclusion of children unable to perform technically acceptable spirometry or complete the exercise challenge may have further affected the representativeness of the final sample. Therefore, the observed EIB prevalence should be interpreted cautiously and may not fully represent the broader population of school-aged children born at ≤32 weeks of gestation.

## 5. Conclusions

This study shows that exercise-induced bronchoconstriction is present in approximately one-third of school-aged children born prematurely and that it may occur independently of classic neonatal risk factors. Our findings support the view that baseline spirometry alone is not sufficient to rule out EIB and that the standard exercise provocation test may be an important component of long-term respiratory follow-up in symptomatic preterm children.

## Figures and Tables

**Table 1:** Demographic, perinatal, and clinical characteristics of preterm-born children (*n* = 42).

Category	Variable	*n* (%)
Demographics	Female sex	27 (64.2)
	Age, years, median (min-max)	7 (7–8)
	Height z-score, median (min-max)	−0.08 (−1.82–2.18)
	Weight z-score, median (min-max)	−0.36 (−2.40–2.18)
	Consanguineous marriage	11 (26.2)
	Gestational week median (min-max), kg	29 (25–32)
	Birth weight, median (min-max), kg	1.23 (0.75–2.35)
	5 min Apgar score, median (min–max)	7 (4–9)
	SGA	8 (19)
	Antenatal corticosteroids	19 (45.2)
	Respiratory distress syndrome	9 (21.4)
	Congenital pneumonia	11 (26.4)
	Neonatal sepsis	28 (66.7)
	Necrotizing enterocolitis	2 (4.8)
	Neonatal PDA	8 (19)
	Neonatal IVH	5 (11.9)
	Neonatal premature apnea	17 (40.5)
	BPD	13 (31)
	Mechanical ventilation	17 (40.5)
	Mechanical ventilation, days, median (min-max)	1 (1–7)
	CPAP	34 (81)
	Oxygen therapy duration, days, median (min-max)	3 (2–61)
	Length of NICU stay, days, median (min-max)	44 (3–93)
	Surfactant administration	9 (21.4)
	Previous hospitalization for lower respiratory tract infection	4 (9.5)
	Breast feed >6 months	23 (54.8)
	Pet in house	2 (4.8)
Personal history of atopy	Asthma	5 (11.9)
	Allergic rhinitis	6 (14.3)
	Eczema history	3 (7.1)
	Food allergy history	2 (4.8)
Family history of atopy	Asthma	6 (14.3)
	Allergic rhinitis	14 (33.3)
	Eczema history	7 (16.7)
Personal history of co-morbidity	Motor developmental problem	7 (16.7)
	Visual problem	9 (21.4)
	Hearing problem	3 (7.1)
	Special education report	2 (4.8)
	ADHD	10 (23.8)
Laboratory	Total IgE, median (min-max), IU/mL	58.4 (5.7–552)
	Eosinophil count/mm^3^, median (min-max)	0.23 (0.1–0.85)

ADHD: Attention-deficit/hyperactivity disorder, BPD: Bronchopulmonary dysplasia, CPAP: Continuous positive airway pressure, IVH: Intraventricular hemorrhage, NICU: Neonatal intensive care unit, PDA: Patent ductus arteriosus, SGA: Small for gestational age.

**Table 2:** Baseline spirometric characteristics, exercise challenge test results, and exercise-related symptoms in the study population.

Variables	*n* (%)
Basal spirometry	
FEV_1_ (% predicted), mean ± SD	89.19 ± 8.48
FVC (% predicted), mean ± SD	82.69 ± 7.83
FEV_1_/FVC (%), mean ± SD	108 ± 7.63
FEF_25–75_ (% predicted), mean ± SD	101.92 ± 23.02
PEF (% predicted), mean ± SD	85.80 ± 14.93
Exercise challenge test results	
EIB positivity at 5 min, *n* (%)	4 (9.5)
EIB positivity at 10 min, *n* (%)	7 (16.7)
EIB positivity at 15 min, *n* (%)	5 (11.9)
EIB positivity at 30 min, *n* (%)	4 (9.5)
Overall, EIB positivity, *n* (%)	13 (31)
ΔFEV_1_ (%) predicted, mean ± SD, at 5 min	5 ± 5.93
ΔFEV_1_ (%) predicted, mean ± SD, at 10 min	5.28 ± 6.12
ΔFEV_1_ (%) predicted, mean ± SD, at 15 min	3.57 ± 7.84
ΔFEV_1_ (%) predicted, mean ± SD, at 30 min	3.21 ± 7.82
Exercise-related symptoms during testing	
Cough	5 (11.9)
Wheezing	2 (4.8)
Dyspnea	16 (38)
Chest tightness	1 (2.4)
Bronchodilator administration after test, *n* (%)	4 (9.5)

**Table 3:** Comparison of perinatal and current clinical characteristics of preterm-born children with and without exercise-induced bronchoconstriction.

Variables	EIB (+) Group, *n* = 13	EIB (−) Group, *n* = 29	OR (95% CI)	*p* Value
Age, median (min-max), years	7 (7–8)	7 (7–8)	2.48 (0.551–11.16)	0.237
Female sex, *n* (%)	11 (84.6)	16 (55.2)	4.46 (0.83–23.85)	0.08
Height z-score, median (min-max),	−0.43 (−1.82–2.18)	−0.06 (−1.72–1.73)	1.08 (0.57–2.04)	0.797
Weight z-score, median (min-max),	−0.43 (−2.4–2.36)	−0.1 (−1.96–2.42)	0.92 (0.57–1.49)	0.757
Gestational week, median (min-max)	31 (25–32)	29 (26–32	1.11 (0.82–1.51)	0.461
Birth weight, median (min-max), kg	1.4 (0.75–2.0)	1.2 (0.8–2.35)	1 (0.99–1.00)	0.470
5 min Apgar score, median (min–max)	8 (5–9)	6 (4–8)	1.72 (0.92–3.22)	0.088
BPD, *n* (%)	2 (15.4)	11 (37.9)	3.36 (0.62–18.08)	0.158
Neonatal premature apnea, *n* (%)	3 (23.1)	14 (48.3)	3.11 (0.70–13.6)	0.1.33
Mechanical ventilation, *n* (%)	3 (23.1)	14 (48.3)	3.11 (0.70–13.68)	0.133
Mechanical ventilation, days, median (min-max)	1 (1–6)	1 (1–7)	0.57 (0.27–1.22)	0.15
Length of NICU stay, days, median (min-max)	37 (10–87)	66 (3–93)	0.98 (0.96–1.00)	0.181
Oxygen therapy duration, days, median (min-max)	1 (1–61)	3 (1–55)	0.98 (0.93–1.02)	0.452
Breast feed > 6-month, *n* (%)	10 (76.9)	13 (44.8)	0.24 (0.05–1.07)	0.062
Diagnosis of asthma, *n* (%)	3 (23.1)	2 (6.9)	0.24 (0.03–1.70)	0.156
Family history of asthma, *n* (%)	2 (15.4)	4 (13.8)	0.88 (0.14–5.53)	0.892
Baseline FEV_1_, (% predicted), mean ± SD	93.23 ± 10.07	87.37 ± 7.14	1.09 (0.99–1.19)	0.054
Baseline FEF_25–75,_ (% predicted), mean ± SD	112.30 ± 27.93	97.27 ± 19.22	1.03 (0.99–1.06)	0.060
Total IgE, median (min-max), IU	39.5 (5.7–194)	60.8 (12.7–552)	0.99 (0.98–1.00)	0.336
Eosinophil count/mm^3^, median (min-max)	0.32 (0.1–0.85)	0.23 (0.07–0.56)	7.68 (0.12–469)	0.331

BPD: Bronchopulmonary dysplasia, NICU: Neonatal intensive care unit.

## Data Availability

The data presented in this study are available upon request from the corresponding author.

## Supplementary Materials

The following supporting information can be downloaded at https://www.mdpi.com/article/10.3390/jcm15186995/s1. Table S1. Comparison of Perinatal and Neonatal Characteristics Between Preterm Children Included and Not Included in the Final Analysis.
