# Supplementary material for: Exercise-Induced Bronchoconstriction in School-Aged Children Born Very Preterm: Prevalence and Associated Clinical Factors
Source: J Clin Med. 2026 Sep 10;15(18):6995. doi: 10.3390/jcm15186995 (PMC13607126; doi:10.3390/jcm15186995)
Supplement: Supplementary file 1 [file jcm-15-06995-s001.zip › jcm-4503273-supplementary.pdf]

Supplementary Table S1. Comparison of Perinatal and Neonatal Characteristics Between Preterm Children Included and Not Included in the Final Analysis

Variables	Included in the final analysis (n = 42)	*Not included in the final analysis (n = 150)	p value
Female sex, n (%)	27 (64.2)	78 (52)	0.216
Gestational week, median (min-max)	29 (25-32)	30 (24-32)	0.83
Birth weight, median (min-max), kg	1.23 (0.75-2.35)	1.28 (0.52-2.15)	0.59
5-minute Apgar score, median (min–max)	7 (4-9)	8 (3-10)	0.058
SGA, n (%)	8 (19)	40 (26.7)	0.420
Neonatal sepsis, n (%)	28 (66.7)	76 (50.7)	0.096
Necrotizing enterocolitis, n (%)	2 (4.8)	13 (8.7)	0.075
Neonatal PDA, n (%)	8 (19)	34 (22.7)	0.772
Neonatal IVH, n (%)	5 (11.9)	26 (17.3)	0.321
Neonatal premature apnea, n (%)	17 (40.5)	70 (46.7)	0.591
BPD, n (%)	13 (31)	39 (26)	0.659
Mechanical ventilation, n (%)	17 (40.5)	55 (36.7)	0.787
Mechanical ventilation, days, median (min-max)	1 (1-7)	1 (0-28)	0.59
Length of NICU stay, days, median (min-max)	44 (3-93)	41.5 (7-240)	0.55

* The group not included in the final analysis comprised children who could not be contacted and those who were contacted but did not meet the study eligibility criteria or could not complete the required assessments.
